# Management of Spinal Metastasis by Minimally Invasive Surgical Techniques: Surgical Principles and Indications—A Literature Review

**DOI:** 10.3390/jcm12165165

**Published:** 2023-08-08

**Authors:** Mikael Meyer, Kaissar Farah, Toquart Aurélie, Thomas Graillon, Henry Dufour, Stephane Fuentes

**Affiliations:** 1 Department of Neurosurgery, La Timone University Hospital, Assitance Publique Hopitaux Marseille, 13005 Marseille, France; mikael.meyer@ap-hm.fr (M.M.); kaissar.farah@ap-hm.fr (K.F.); thomas.graillon@ap-hm.fr (T.G.); henry.dufour@ap-hm.fr (H.D.); 2 Spine Unit, La Timone University Hospital, Assitance Publique Hopitaux Marseille, 13005 Marseille, France; aurelie.toquard@ap-hm.fr; 3 Department of Orthopedic Surgery, La Timone University Hospital, Assitance Publique Hopitaux Marseille, 13005 Marseille, France

**Keywords:** minimally invasive, spinal metastasis, surgical management, spinal instability neoplastic metastatic spinal disease, oncology, minimally invasive spine surgery, neuronavigation

## Abstract

Background: Spinal metastasis is becoming more frequent. This raises the topics of pain and neurological complications, which worsen the functional and survival prognosis of oncological population patients. Surgical treatment must be as complete as possible in order to decompress and stabilize without delaying the management of the oncological disease. Minimally invasive spine surgical techniques inflict less damage on the musculocutaneous plan than opened ones. Methods: Different minimally invasive techniques are proposed in this paper for the management of spinal metastasis. We used our experience, developed degenerative and traumatic pathologies, and referred to many authors, establishing a narrative review of our local practice. Results: Forty-eight articles were selected, and these allowed us to describe the different techniques: percutaneous methods such as vertebro/kyphoplasty, osteosynthesis, mini-open surgery, or that through a posterior or anterior approach. Also, some studies detail the contribution of new technologies, such as intraoperative CT scan and robotic assistance. Conclusions: It seems essential to offer a lasting solution to a spinal problem, such as in the form of pain relief, stabilization, and decompression. Our department has embraced a multidisciplinary and multidimensional approach to MISS, incorporating cutting-edge technologies and evidence-based practices.

## 1. Introduction

Spinal metastases have an increasing prevalence and are responsible for pain and neurological complications that aggravate the prognosis of this population of oncological patients [1]. The median survival of patients with spinal metastasis is approximately 9 months. There is no formal, predictive factor of the good or bad progression of a patient at a specific time [2]. Prognostic scores have been described in various reports, including the Tokuhashi index (and the modified Tokuhashi index) and Tomita’s classification [3,4], which lead to a decision being made of whether or not a surgical treatment is necessary.

However, a meta-analysis revealed that these indices alone are not sufficient to decide on a treatment; they should be one part of a multidisciplinary and personalized approach [5]. Nowadays, there are very few indications for brace treatment [6]. Its main indications are probably a multifocal spinal involvement, which will be treated with radiotherapy, or a patient with a very short life expectancy (<3–6 months).

Minimally invasive spine surgery (MISS) has revolutionized the field of spinal interventions, especially in spinal tumors offering patients less postoperative pain, reduced recovery times, and improved surgical outcomes [7]. They have shown identical efficacy to open surgeries in degenerative and traumatic surgery [8,9,10,11]. In our department, we have embraced cutting-edge technologies and advanced techniques to provide the highest standard of care for patients with various spinal tumoral pathologies. This narrative review aims to highlight the key aspects of our local practice in MISS, including the use of SINS score, neurological evaluation, navigation, perioperative CT scan, and robotic assistance.

## 2. Material and Methods

This narrative review was approved by an IRB (n° IRB00011687). Each patient case was discussed with a multidisciplinary staff member, such as a neurosurgeon, oncologist, radiotherapist, or radiologist.

The management decision is based on patient clinical statute (World Health Organization score, neurological assessment, pain), the oncological project, survival expectancy, and radiological data. Neurological evaluation is an integral part of our preoperative and postoperative assessments. We utilize standardized scoring systems to evaluate the motor and sensory functions of patients. This aids in identifying potential neurological deficits and helps us to monitor improvements following surgery. The spinal instability neoplastic score [12] (SINS) plays a pivotal role in the evaluation of patients with spinal instability related to neoplastic conditions. This scoring system helps us categorize and manage cases effectively, ensuring a tailored approach is taken towards each patient. By considering various factors such as pain, spinal alignment, bone lesion location, and vertebral body collapse, we can assess the degree of instability and decide on the most suitable treatment option. The proposition of management is discussed with the patient and their family. If the SINS score is ≤6, the spinal lesion is considered stable, and besides systemic therapy and/or radiotherapy, some surgical approaches can present an alternative to reduce pain and painkiller consumption.

Among these approaches, vertebroplasty or thermoablation are standard use in our regular practice.

### 2.1. Vertebral Augmentation Techniques: Vertebroplasty or Balloon Kyphoplasty

Vertebroplasty was first described in 1987 by Galibert et al. for the treatment of vertebral angiomas, C2 in particular [13]. The indications were then extended to osteoporotic fractures and spinal metastasis [14,15]. The aim is to inject a PMMA (polymethylmethacrylate) radiopaque cement into the vertebral body through a trocar.

Our department utilizes image-guided techniques to inject bone cement directly into the fractured vertebra, stabilizing the bone and providing rapid pain relief. It minimizes hospital stays and allows for a quicker return to daily activities when possible.

Surgery is performed under sedation and local anesthesia, or under general anesthesia. The whole spine can be eligible. The approach is generally posterior for thoracic and lumbar spine involvement through transpedicular, unilateral, or bilateral approaches. In some cases, especially from C2 to C7, anterior approaches through direct access to the vertebral body can be performed.

The indication of vertebroplasty is the treatment of pain caused by a lesion of the vertebral body. However, there are contraindications: medullary compression, rupture of the posterior wall, compression >75%, recent fracture, increasing the risk of intra-ductal leakage, this leakage being the main complication of vertebro/kyphoplasty (9.2 to 78.5% depending on the series). They can be responsible for mechanical complications, such as compression of the nervous tissues: marrow, root, but also pulmonary embolism [16]. This risk of leakage varies according to the pathology; it is more important in case of neoplastic lesion. However, only 3.4% of root lesions are found in the treatment of vertebral metastases with cement leakage.

The balloon kyphoplasty technique was developed to mitigate the risk of the leakage of cement caused by vertebroplasty [17].

The principle is the same: injection of PMMA cement into the vertebral body after having elevated the upper vertebral plateau with the aid of a balloon. The goal is to create a cavity in the vertebra to inject a thicker cement, which reduces the risk of leakage. The effect of the balloon also has a mechanical action to reduce vertebral compression. The only absolute contraindication is medullary compression, the rupture of the posterior wall being a relative contraindication, because the risk of leakage is limited. Sometimes a stent can be set up before the cement injection to maintain the vertebral height and prevent the superior endplate from collapsing. But their indication is not recognized in oncology. In his meta-analysis, Papanastassiou found 41% of leakage in patients treated with vertebroplasty versus 18% for kyphoplasty [18].

In some cases, when vertebroplasty or kyphoplasty are challenging, such as in upper cervical spine, the use of a perioperative CT scan has become routine in our department for these cases. This approach provides invaluable insights into patient anatomy and helps us visualize the surgical site with exceptional clarity. Intraoperative CT scans can be performed to confirm the accuracy of instrument placement and assess surgical outcomes, ensuring that any discrepancies are addressed promptly. In addition, robotic-assisted MISS is another area where our department has made significant progress [19]. Utilizing robotic systems for spinal surgeries offers several advantages, including enhanced precision, reduced radiation exposure, and improved patient safety [20].

**Figure 1:**
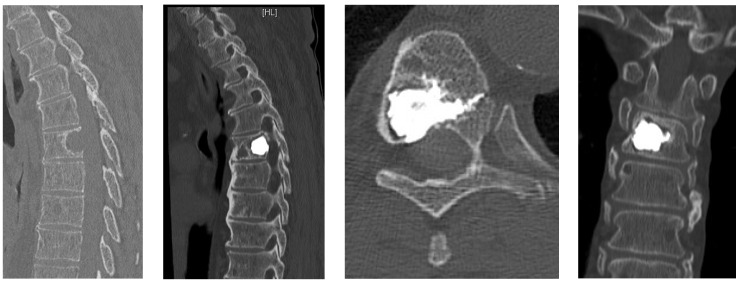
T8 kyphoplasty with intraoperative CT scan planning.

**Figure 2:**
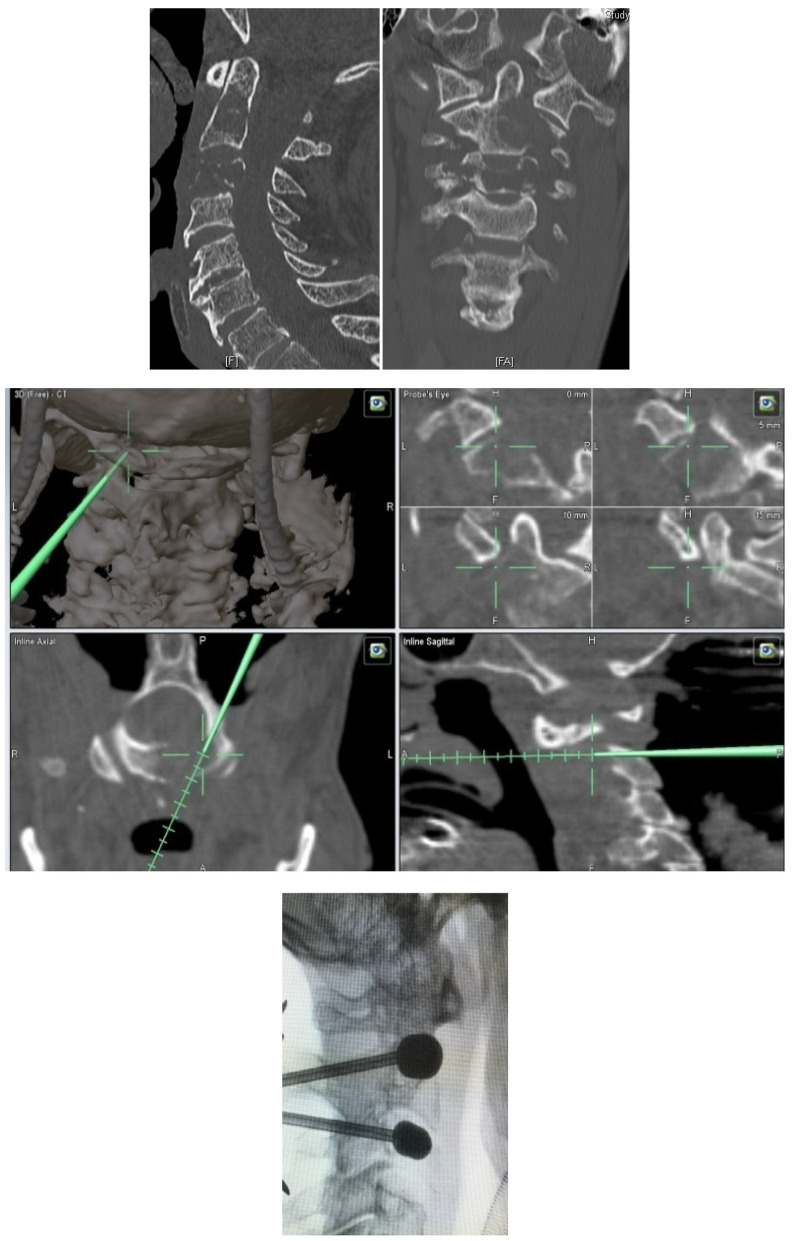
C2 and C3 kyphoplasty with intraoperative scan planning.

**Figure 3:**
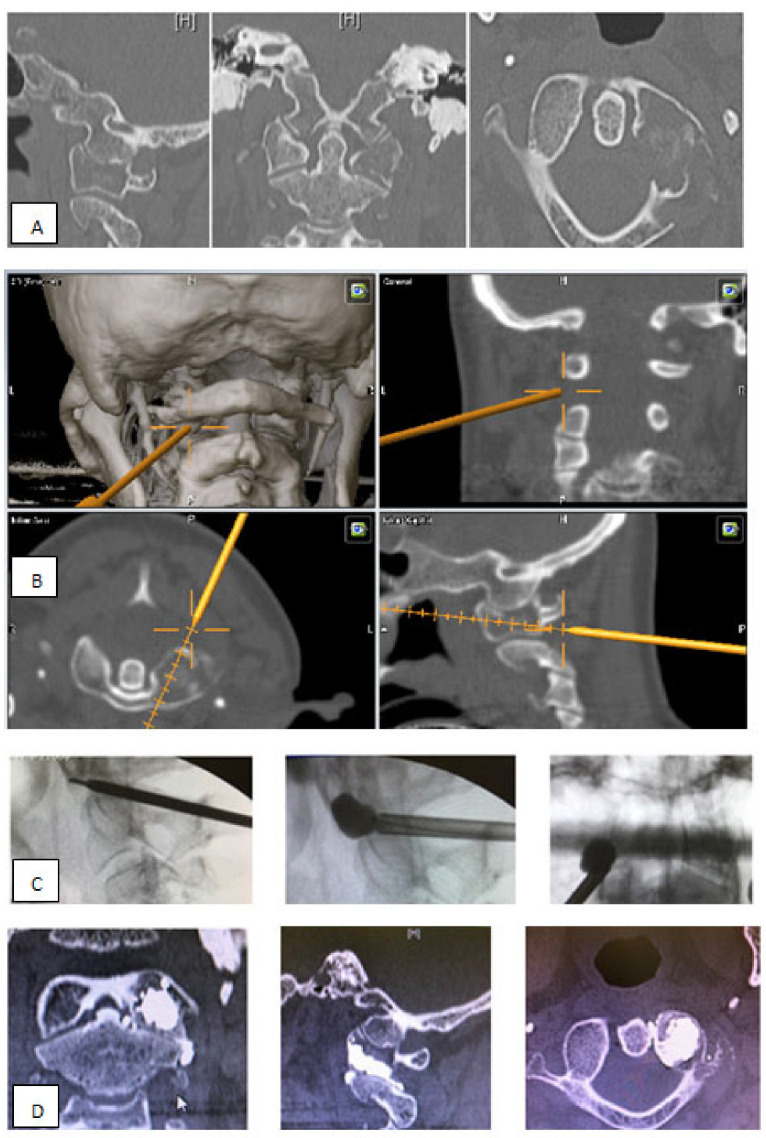
C1 kyphoplasty with intraoperative scan planning. (**A**) Sagittal, B axial and C coronal ct scann slice. Osteolysis of the left lateral mass of the atlas; (**B**) The navigated jamshidi into C1 lateral mass; perioperative screeshots showing real time 3D, coronal, axial and sagittal; (**C**) Intraoperative fluoroscopy; (**D**) Control scan; A. sagittal, B. axial and C. coronal reconstruction: bone windowing showing the filing of the lateral mass without intracanal leakage.

Intraoperative planning, particularly in MISS, provides:

- Unrestricted reconstruction of datasets with views along the path of the needle with planning in 3D and 2D reconstructions (Figure 1).
- Decreased X-ray exposure for the MISS technique. Navigation enables a reduction in X-ray exposure to the surgical team and the patient. Through a real time visualization of instruments, skin incisions and trajectories can be planned [21].
- Posterior percutaneous kyphoplasty for cervical spine metastases (Figure 2); case of C2-C3 kyphoplasty.

- Metastatic lesions of C1 are extremely rare, and their treatment by percutaneous cement augmentation is considered to be a technically challenging procedure due to complex anatomy. We also performed percutaneous kyphoplasty in a painful osteolytic lesion located on the left lateral mass of C1 through a posterolateral approach using a 3D CT scan intra-operative navigation system and fluoroscopy (Figure 3) [22].

Under certain conditions, especially when SINS score is >13 or even sometimes >6, percutaneous kyphoplasty or vertebroplasty may be associated with percutaneous osteosynthesis for two purposes:

- Maintaining spinal stability;
- Optimally reducing pathological fracture. The reduction by osteosynthesis will then allow for optimal vertebro/kyphoplasty.

### 2.2. Osteocool Radiofrequency

The ablation of targeted tumor(s) was performed with the OsteoCool RF Ablation System (Medtronic, Dublin, Ireland). The system consists of an RF generator, peristaltic pump, and the connector hub, which provides 2 channels for the use of the ablation probes and 2 channels for the use of the optional independent thermocouples. The bipolar ablation probe is a coaxial, bipolar technology that provides localized tumor ablation and automatically adjusts the power to keep the RF heating within the desired treatment range. The active tip of the RF ablation probe is internally cooled with circulating sterile water in a closed-loop system. RF energy heats the tissue while circulating water moderates the temperature in close proximity to the active tip to minimize charring. The ablation volume and time are determined based on the probe tip size.

The procedures were performed with computed tomography or fluoroscopic guidance under general anesthesia per the institution’s standard of care. The target tumors were accessed by using an 8- or 10-gauge introducer cannula. In the thoracolumbar spine, the vertebral bodies were accessed via a transpedicular or parapedicular approach. The ablation probe lengths were predetermined and placed through the access cannula. The RF ablation protocol was performed by using the preset manufacturer algorithm. At the completion of RF ablation, PMMA, if used, was injected through the same bone access cannula.

## 3. Osteocool

More recently, several small observational studies of percutaneous radiofrequency (RF) ablation have demonstrated pain relief (mood and pain intensity improvement) and decreased opioid agent use [23,24,25,26,27,28].

### 3.1. Osteosynthesis

Historically, percutaneous osteosynthesis was suggested as a complement to an anterior approach in degenerative spinal diseases [29]. Currently, it is very common in traumatology and even represents the management of first intention in non-neurological spinal fractures and/or in patients with polytrauma [30,31,32]. The technique is well codified and performed under general anesthesia on a table dedicated to radiopaque spinal surgery under fluoroscopic control. The sequence is generally identical regardless of the various laboratories of osteosynthesis: introduction into the pedicles of a trocar and then a spindle and finally the cannulated screw. There are ancillaries of reduction (distraction, lordosis, iron to bend) that are equivalent in power and results to open techniques [33]. These ancillaries and modern radiological means make it possible to carry out osteosynthesis from C0 to the pelvis.

**Figure 4:**
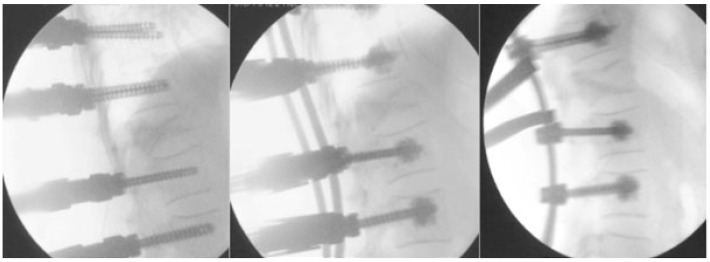
Augmented screws.

This technique of percutaneous screwing ensures one rate of good position of the implants in more than 90% of cases. In the study of Vanek et al., only 2 out of 72 patients had one or more overly medial screws or 2.8% versus 5 out of 68 for the conventional technique (7.4%) [34]. The risk in this population of elderly patients who were osteoporotic with multiple bone lesions is the mechanical failure of the assembly. A solution could come from the use of percutaneously implanted increased screws. The use of augmented screws (Figure 4) makes it possible to reduce the risk of tearing and mechanical failure of fragile bone assemblies: osteoporotic or multi metastatic [16,35]. They follow strict rules of use, and surgical planning is essential to the use of each vertebra of a screw of suitable diameter and length to avoid complications. The quantity of cement to be injected is, at most, two milliliters per screw (it depends on the level of screwing with a minimum of 1.2 milliliters).

In the literature, the risk of leakage is frequent, ranging from 5 to 39%. In Pesenti et al.’s study, only one patient out of twelve presented with some complication, such as pulmonary embolism on cement embolus (8.3%) [16]. Thanks to the quality of the screw upholding, in situ reductions could be made.

In some cases, when neurological elements (spinal cord and/or roots) suffer, decompression is needed, and this can be performed through MISS techniques.

### 3.2. Open Ways Mini Invasives

#### 3.2.1. Posterior Approach

All percutaneous procedures are not recommended for patients with symptomatic spinal cord compression that justifies aggressive decompression surgical management. Patchell et al. showed the value of using surgical decompression in patients with spinal cord compression caused by spinal metastasis in a multicenter randomized study [36]. Out of 50 (84%) patients, 42 were walking after surgery + radiotherapy, versus 29 out of 51 patients (57%) in the radiotherapy group alone. These results are significant. Posterior minimally invasive actions are based on the use of tubular spacers, widely used in degenerative spinal surgery: lumbar disc herniation, narrow lumbar or cervical canals, spondylolisthesis, and more recently for the removal of intradural extramedullary tumors [37,38]. They proved that by reducing the muscular dilapidation, the rate of infection was decreased, as well as hematoma and postoperative pains [39]. The improvement in minimally invasive techniques allows laminectomies, arthropediculectomies, and therefore high-quality spinal decompression to be performed by using these spacers.

In 2012, Zairi et al. reported their prospective study of 10 patients with symptomatic thoracic or lumbar metastases (pains, root, or neurological signs) [40]. Transpedicular vertebrectomy, decompression, and percutaneous stabilization were performed. The technique of percutaneous pedicular screwing two levels above and two below was the first step, then decompression occurred under a tubular retractor. The operative time was of 170 min with blood losses of 400 milliliters, but no blood transfusion was necessary. The average length of stay was 6 days. In total, 80% of the patients recovered to at least one grade of the Frankel score, and the EVA fell from 5.5 to 2. All patients underwent postoperative radiotherapy in a short timeframe of about 3 weeks.

By minimizing surgical morbidity, minimally invasive decompression techniques are ideally suited to this type of patient. They make it possible to link more quickly the treatment of the primary lesion by radiotherapy and chemotherapy. They may be associated with vertebro/kyphoplasty and/or percutaneous osteosynthesis, as described by Joseph H Schwab [41]. Twenty-four patients with thoracic or lumbar metastases without medullary compression were treated with percutaneous osteosynthesis, and one patient underwent minimally associated invasive decompression due to L5 radiculalgia. For the record, seven patients who could not walk preoperatively because of the pain were walking after surgery. On the third postoperative day, EVA had significantly decreased. The main objective of this study was to show the major interest of minimally invasive surgery aiming at improving the quality of life of patients (pain, mobility).

#### 3.2.2. Minimally Invasive Anterior Approaches

The aim of anterior surgery is twofold: to resect a corporal tumor mass and/or to ensure the mechanical strength of the spine by reinforcing the anterior column by a corporectomy implant. Anterior spinal surgery can be divided into three entities: T3 to T10, T11 to L2, and L2 to L5. In all cases, the incision is traced after scopic locating around the vertebra to reach.

#### 3.2.3. Thoracotomy

An opening of 3 to 5 cm is made. Resection of the rib on the first approach reduces intercostal muscle decay. The use of a tubular retractor makes it easier to access and spread the planes. The use of video endoscopy allows a comfort of lighting and vision facilitating the procedure [42]. Conventionally, it is carried out by an easy and rapid trans pleural route, but it often requires pulmonary unilateral ventilation. The retropleural route can be performed without pulmonary unilateral ventilation since the opening of the pleura is avoided, but it is more difficult to perform [43,44]. In 2005, Tsung-Jen Huang reported that in his experiment on 46 patients with thoracic metastasis between T3 and T12 [45], 29 were operated on using an anterior minimally invasive procedure (screws, plaque, and cement only), and 17 were operated on via standard thoracotomy. There was neither a significant difference in blood loss and operative time nor in the neurological recovery, which was 1.2 grade of the Frankel score on average in the two groups. On the other hand, there was a significative difference in postoperative follow-up: only 6.9% of patients in the MIS group required intensive care, compared with 88% of patients in the standard thoracotomy group, which represents an enormous improvement in its weaker patients.

#### 3.2.4. Retroperitoneal Retropleural Lumbotomy (Figure 5)

From T11 to L2, the most widely used technique is retropleural/retroperitoneal minimally invasive through the 10th, 11th, or 12th rib. It is a left approach, in right lateral decubitus. The cutaneous incision and the costal resection are performed as described for the thoracotomy. The section of the left posterior diaphragmatic pillar makes it possible to put the retroperitoneal space in communication with the retropleural space [46].

**Figure 5:**
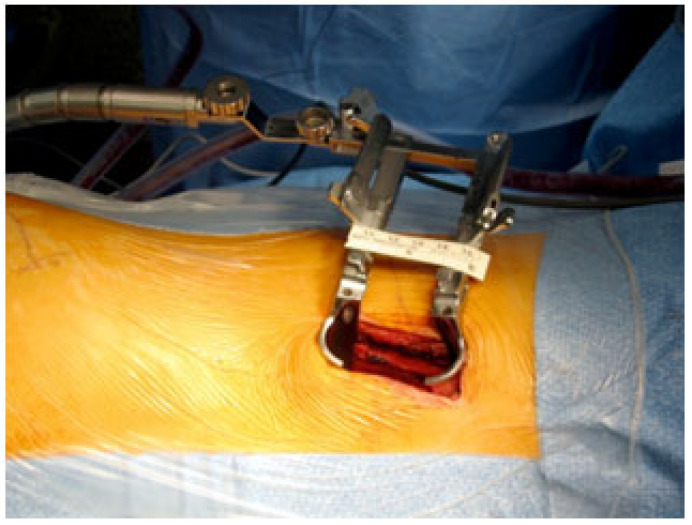
Minimal Invasive anterior surgical approach.

#### 3.2.5. Classical Lobotomy

Under L2, we favor the classic retroperitoneal approach described by Mehren et al. With a right lateral decubitus installation, an incision of 4 to 6 cm centered on fluoroscopic control and an approach in dissection of the three muscular planes provides access to the spine without significant muscular damage [47]. Corporal reconstruction is then indispensable. There is no consensus for the type of equipment. The most commonly used are telescopic or non-telescopic cylindrical coronal prostheses made of titanium, filled with PMMA cement, or corporal prostheses in Peek.

**Figure 6:**
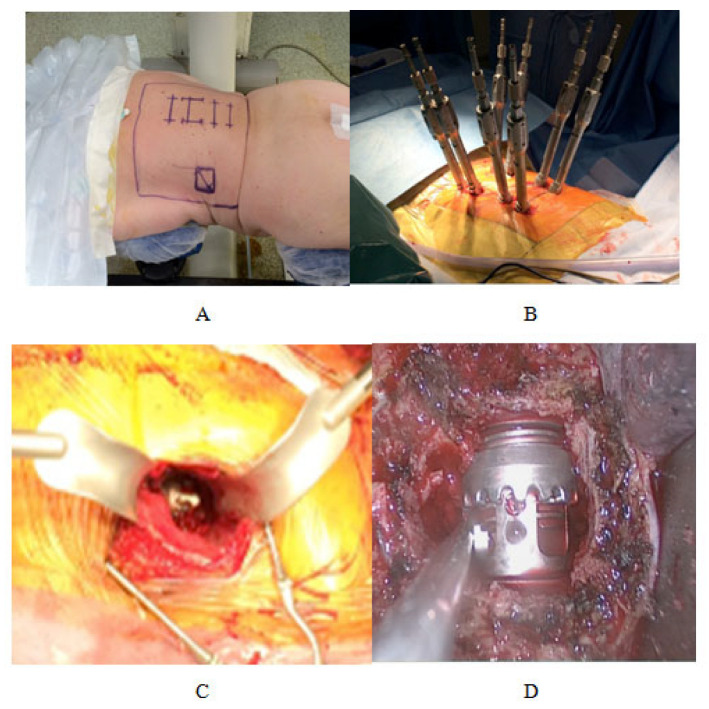
Minimally invasive corporeal resection associated with posterior percutaneous osteosynthesis. (**A**) Decubitus ventral Installation; (**B**) Percutaneous osteosynthesis; (**C**) Minimal invasive Anterior approach; (**D**) Corporeal prostheses (titanium).

The surgeon can perform this minimally invasive corporeal resection alone or associate it with posterior percutaneous osteosynthesis, which would ensure a better stabilization of the mounting [48]. This circumferential osteosynthesis can be performed in a single operative time, the patient being placed in the ventral decubitus (DV) for both approaches (Figure 6). A single field is made and includes the two incisions.

Minimally invasive anterior surgery is thus a surgical tool adapted to these patients when a corporeal reconstruction is necessary. This technique can be coupled with other minimally invasive techniques (osteosynthesis or decompressive). They can be performed in a single surgical time if necessary because it is not hemorrhagic, which then ensures a complete treatment of a vertebra, whether in the ventral or lateral decubitus.

## 4. Discussion

In recent years, the management of spinal tumors has seen significant advancements, with MISS emerging as a promising technique. As part of our department’s ongoing efforts to provide the best possible care to our patients, this narrative review explored the role of MISS in the management of spinal tumors. The advantages, inconveniences, and limitations associated with this study will be discussed to better understand its potential impact on patient outcomes and our routinely activity.

When talking about MISS, many advantages are to be reported. Some of the most significant are smaller incisions, reduced muscle damage, and less blood loss. This can lead to faster recovery, shorter hospital stays, and decreased postoperative pain for patients. They often experience a quicker return to daily activities and work compared to traditional open surgery [49,50,51].

The reduced trauma to surrounding tissues facilitates a faster healing process, allowing patients to resume their normal routines sooner. With smaller incisions and less exposure of tissues, the risk of surgical site infections is typically lower in MISS compared to open surgery. This can positively impact patient outcomes and reduce the need for prolonged antibiotic use [52].

Moreover, intraoperative CT scan and robotic assistance allows access to what is considered challenging locations, such as the upper cervical spine, due to surrounding elements (vertebral artery and spinal cord) [19].

It is advocated to mainly reduce the risk of neurovascular injury. Securing routing procedure placement under navigation guidance offers also the possibility of inserting material via a percutaneous approach with reduced radiation exposure for patients, the surgical team, and operative room staff. Unlike percutaneous kyphoplasty performed by a radiologist, ergonomic is more appreciable in an OR with a 3D navigation system as the donut of the iCT is removed from the working area after image acquisition.

The employment of robotics systems has increased in the field of spine surgery given that they provide precise, reliable, and effective procedures that can be performed quickly. In combination with a navigation system, robotics systems theoretically promise more accurate pedicle screw fixation and less soft tissue damage. Pedicle screw fixation is a representative application of robotics systems. Although there are insufficient clinical data regarding the utility of robotics systems in spine surgery, several studies have reported that the accuracy of pedicle screw fixation by robotics systems is superior to that of free-hand as well as fluoroscopy-guided pedicle screw fixation. One of the most important advantages of robotic spine surgery is that it can overcome the mental and physical fatigue of the surgeon during the procedures, which could provide better clinical and surgical outcomes. Unfortunately, robotics systems are rarely applied due to their high cost [53,54].

It is also quite possible to combine several MISS techniques to adapt to patients’ demands, especially pain, neurological assessment, and radiological findings.

**Figure 7:**
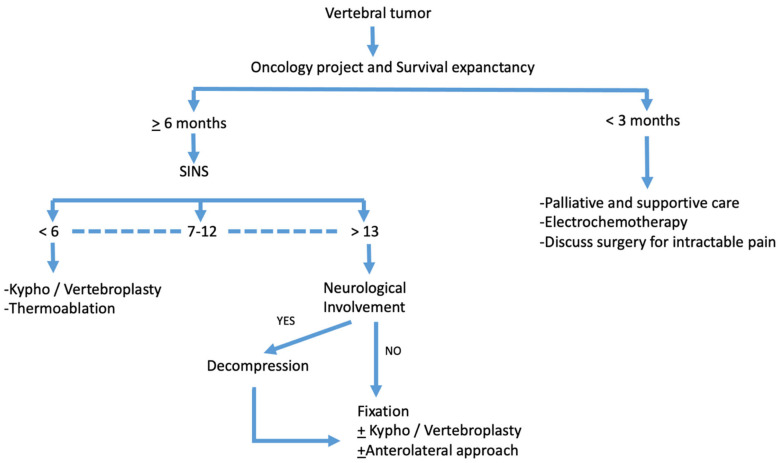
Decision algorithm.

Therefore, Figure 7 presents an overview of our main decision algorithm for patients harboring spinal tumors.

Due to the advancement of systemic therapy, many patients with spine metastasis live longer. Thus, surgery for spinal metastasis is subject to prior chemotherapy or prior radiotherapy, adding to the patients’ medical fragility in addition to all the risks of non-oncological spine surgery. Optimizing surgery for patients with spine metastasis is essential to prevent postoperative morbidity. With technological advancement, MISS for spine metastasis has become feasible, and thus has been considered a new treatment option over traditional open surgery. Several studies have reported equivalent functional outcomes and faster recovery with reduced morbidity, such as blood loss and surgical-site infection [55,56,57,58].

Meanwhile, MISS can present some inconveniences.

First, cost and equipment: Incorporating MISS into a surgical practice may require significant initial investments in specialized equipment and training. While the potential benefits may outweigh the costs in the long run, this can pose financial challenges for smaller medical centers or clinics.

Secondary, procedure time: Some MISS procedures may take longer to perform compared to traditional open surgery. The need for meticulous precision and the use of specialized instruments can extend the surgical duration, potentially affecting operating room schedules.

Lastly, radiation exposure: In certain cases, MISS procedures may involve the use of intraoperative imaging guidance, exposing patients and surgical staff to higher levels of radiation. While efforts are made to minimize exposure, especially for the surgical staff, this remains a concern that should be considered and managed appropriately [59].

Finally, this narrative review exploring the role of MISS in the management of spinal tumors presents some limitations.

We did not present any clinical data regarding pain scale scores, painkiller consumption, or neurological involvements. Our only aim was to report diverse MISS techniques used in our daily practice for our patients. A follow-up period and discussion of the complications also lack.

Figure 7 represents a comprehensive decision algorithm in the management of spinal tumors within our institution in accordance with the current guidelines.

## 5. Conclusions

Patients with spinal metastases are frail, older, and have a limited life span. Therefore, it seems essential to offer a lasting solution to a spinal problem: pain relief, stabilization, and decompression. This spinal injury can greatly compromise the patient’s life quality because of the pain and neurological disorders that can occur. Our department has embraced a multidisciplinary and multidimensional approach to MISS, incorporating cutting-edge technologies and evidence-based practices. By implementing the SINS score, comprehensive neurological assessments, navigation technology, perioperative CT scans, and robotic assistance, we aim to provide the best possible outcomes for our patients. As advancements in MISS continue, our commitment to delivering exceptional care remains unwavered.

## Data Availability

Data are available by request to the author.
